# Sensory Habituation as a Shared Mechanism for Sensory Over-Responsivity and Obsessive–Compulsive Symptoms

**DOI:** 10.3389/fnint.2020.00017

**Published:** 2020-04-03

**Authors:** Tamar Y. Podoly, Ayelet Ben-Sasson

**Affiliations:** ^1^Department of Occupational Therapy, Faculty of Social Welfare and Health Sciences, University of Haifa, Haifa, Israel; ^2^Cognetica: The Israeli Center for Cognitive Behavioral Therapy, Tel Aviv, Israel

**Keywords:** sensory, habituation, OCD, adults, electrodermal activity

## Abstract

**Background:**

Some individuals who suffer from obsessive–compulsive (OC) disorder (OCD), report disturbing sensory preoccupations. The inability to stop obsessing over stimuli resonates with a difficulty in sensory habituation. Impaired sensory habituation, to a degree that clearly dysregulates response to sensory stimuli, and impairs participation in everyday activities, can be part of a disorder known as sensory over-responsivity (SOR). Although previous studies indicated a correlation between OCD and SOR, physiological experiments show that individuals with OCD are not more sensitive to sensory stimuli than controls. In the current study, we (1) validated a sensory habituation psycho-physiological protocol and (2) tested whether a “slow to habituate” mechanism can explain the occurrence of elevated SOR and OC symptoms.

**Methods:**

We designed a protocol to test auditory sensory habituation through electrodermal activity (EDA) recording. The protocol included two randomly ordered aversive and neutral sound conditions; each set of six everyday life sounds was presented as a continuous stimulus. During the presentation of sounds, EDA was measured and participants could press a button to shorten the stimuli. Participants also completed sensory and OC symptom questionnaires. Participants included 100 typically developing adults that were divided into high versus low OC symptom groups. Mixed models analysis was used throughout to meet the need for capturing the temporal nature of habituation.

**Results:**

Distinct physiological indices were computed to measure sensitivity versus habituation. Habituation was slower in the aversive versus neutral condition. Sensitivity was higher for the aversive stimuli. Self-report of sensory habituation and sensitivity partially correlated with the physiological habituation indices. A comparison of the physiological pattern between those with high versus low OC symptoms revealed significant differences in the habituation and sensitivity indices, across conditions.

**Conclusion:**

The interplay between SOR and OC symptoms can be explained by a “slow to habituate” mechanism. Identifying behavioral and physiological markers of sensory problems in OCD is important for assessment, intervention and the discovery of underlying mechanisms.

## Introduction

Sensory abnormalities in obsessive–compulsive (OC) disorder (OCD) have received less attention in the OCD literature than cognitive aspects. Descriptions, reports, and studies about sensory abnormalities in OCD have increased only recently (Grimaldi and Stern, 2017). Among the various sensory abnormalities described in the OCD literature, extreme sensory over-responsivity (SOR) is noted most often (Conelea et al., 2014; Lewin et al., 2014; Taylor et al., 2014). In addition, SOR is the most impairing form of sensory modulation disorder (SMD) regardless of OCD (Ben-Sasson et al., 2009). Irritating sensations reported in OCD include strong aversion to the odor of certain foods; inability to endure innocuous sounds, such as breathing, rubbing, or sniffing; high sensitivity to noise; and intolerance for clothing or different textures. The current investigation explores two potential mechanisms, atypical sensitization and habituation, that can explain the high rates of SOR in OCD. Whereas SOR is generally inferred to reflect an atypical sensitization level (i.e., lower response threshold), in fact it may be a factor of prolonged habituation (i.e., longer duration of response).

From physiological and neurological perspectives, *habituation* is a parallel process to sensitization and is described in the dual-process theory (Groves and Thompson, 1970). Habituation is a decremental process, whereas *sensitization* is incremental, enhancing the tendency to respond. Thus, when habituation exceeds sensitization, habituation dominates, and vice versa. These processes occur simultaneously, and the behavioral output reflects a summation of both. The reaction decrement or increment can be detected at the cellular and synapse levels (Thompson, 2001), the central nervous system level (Gudin, 2004), and in specific brain areas, such as the amygdala and the hippocampus (Breiter et al., 1996).

From a sensory modulation perspective, sensitization and habituation are dimensions on a continuum of a neurological threshold. This threshold indicates how intense the stimulation must be for the individual to notice it and falls on a continuum from low to high (Dunn, 1997). We hypothesize that high sensitivity to stimuli, along with difficulty habituating to them over time, might be present in SOR. What is the relation between sensitivity and habituation? High responsivity may cause a slower habituation process; however, if the central nervous system does not habituate effectively, it can create a higher sensitivity. This assumption has guided our examination of the association between slow habituation and reported SOR.

Habituation, the most basic form of learning, is a decrease in the intensity of response to a specific stimulus following prolonged exposure to it (Peeke and Petrinovich, 1984). It allows people to reflexively filter irrelevant information and to focus on significant stimuli. The habituation process has a crucial role in forming a modulated sensory response. Despite this, habituation has not been sufficiently studied in the context of SMDs. This study aimed to develop procedures for quantifying habituation and to understand its correspondence with self-reported SOR symptoms and with the more studied dimension of SOR, sensitization.

The literature presents evidence for the unique clinical co-existence of SOR and OCD (Rieke and Anderson, 2009; Conelea et al., 2014; Lewin et al., 2014). Researchers point to a specific sensory OCD subtype. It is characterized by male predominance, a clinical course that is more aggressive (i.e., greater number of obsessions and compulsions and shorter times between each onset), high comorbidity with Tourette’s syndrome and tic disorders, and more ritual repetition and tic-like compulsions (Rosario-Campos et al., 2001; Fontenelle et al., 2003; O’Leary, 2005). The symptoms of this specific sensory OCD subtype were also found to be related to sensory-based obsessions and compulsions (Miguel et al., 2000; Prado et al., 2008). Studies showed that in the sensory OCD subtype, individuals report having sensory-like compulsions without obsessions preceding them (Leckman et al., 1993; Coles et al., 2003). These correlations can also be found with respect to traits in typical populations. For example, correlations between repetitive or ritualized behaviors and SOR have been shown in both typical and clinical samples (Bart et al., 2017; Di Renzo et al., 2017).

From a clinical perspective, it is well understood how high sensitivity to noises, smells, or tactile stimuli can lead to avoidance and withdrawal from specific situations that involve sensory stimuli experienced as aversive. Another way of explaining this interplay is from the incompleteness perspective (Summerfeldt, 2004), which is a cognitive core dimension of OCD. Some actions or sensations “do not feel right” (e.g., both shoelaces are not tied with exactly identical tension or the hair is not parted exactly in the middle). People with SOR or with OCD may report sensations of incompleteness (Dunn, 1997; Ecker et al., 2014). This association strengthens the possibility that SOR affects the obsessive tendency in OCD, as well as the compulsion aspect of the disorder. The correlations presented in previous studies did not distinguish between different SOR dimensions, sensitivity and habituation, dimensions the current study put forward to investigate.

When considering sensory habituation as an underlying mechanism to explain sensory symptoms of OCD, the resemblance in symptoms is striking. Individuals who are slow to habituate tend to pay attention to a stimulus continuously, even long after it was presented. It is no wonder that these individuals might become obsessed with the perception of that stimulus or even try to avoid it. They might engage in behaviors intended to reduce the distress and discomfort that arise from encountering the sensory stimulus. These behaviors can evolve and fixate as compulsions (Summerfeldt, 2004). Some studies examined whether individuals with OCD have lower neurological thresholds and are therefore more sensitive to stimuli. However, those studies found no differences between tactile and olfactory stimuli-detection thresholds of individuals with OCD and those of healthy controls (Belluscio et al., 2011; Güçlü et al., 2015). Moreover, the subjective intensity near threshold was similar to normal, despite noting that faint stimuli were generally more bothersome (Belluscio et al., 2011). The investigators suggested that the problem is not that of simple sensory perception but might be a deficiency of habituation (Hallett, 2015). Understanding sensory habituation in relation to OC symptoms is one of the aims of this study.

Audition is the most commonly reported bothersome modality among individuals with SOR (Royeen and Fortune, 1990; Ben-Sasson et al., 2009). Although other sensory modalities, such as tactile and olfactory, were found to be involved in OCD (e.g., Güçlü et al., 2015), our study focused on the auditory modality because it is often observed in OCD (Buhlmann et al., 2007; Buse and Roessner, 2016) and it is feasible to quantify in an experimental design. Auditory SOR can be expressed by sensitivity to specific sounds (e.g., breathing and electronic devices), background noises (e.g., air conditioner and people talking), or sensitivity to the intensity of the sound (e.g., loud tones and noisy environments). Some researchers described a selective-sound sensitivity syndrome (also known as misophonia) as a comorbidity of OCD or a specific case of OCD (Neal and Cavanna, 2013; Webber et al., 2014). Misophonia, in particular, can be viewed as an extreme display of SOR. Examples of atypical auditory processing in patients with OCD can be found in the tendency for higher electromyogram heart rate responses to loud tones and the slower decline in electrodermal activity (EDA) after stimulus presentation found (Buhlmann et al., 2007).

Auditory habituation is depicted by the theoretical construct of sensory gating (SG), which describes the process of filtering irrelevant auditory stimuli from all possible environmental stimuli in the central nervous system (Cromwell, 2008). In this process, irrelevant auditory stimuli are ignored while other more relevant input is obtained simultaneously (Kisley et al., 2004; Davies et al., 2009). SG has a major role in modulating sensory stimulus at the neurological level. It is in fact responsible for the inhibition process that occurs when habituating to a non-relevant stimulus. Auditory startle response, which is an index reflecting SG, was found to be less inhibited in individuals with OCD (Swerdlow et al., 1993; Hoenig et al., 2005; Ahmari et al., 2012).

Sensory abnormalities are commonly measured using self-report questionnaires. These instruments mostly inquire about behavioral responses to stimuli from different sensory modalities, but some also describe emotional responses to sensory stimuli. Few self-report questionnaires focus on the perceptual or temporal aspects of SOR (e.g., Tavassoli et al., 2014; withheld for blind review). Contrary to the wealth of research on behavioral sensory self-report measurements, research with physiological measurements, specifically those measuring sensory habituation, is scarce.

A few studies have used physiological methods to measure SOR, for example, EDA (McIntosh et al., 1999), electroencephalogram, prepulse inhibition (Davies and Gavin, 2007; Davies et al., 2009), and cardiac vagal tone index (Schaaf et al., 2003). The sensory challenge protocol (McIntosh et al., 1999) has been used to systematically record physiological responses to sensations. This protocol was created to gauge individuals’ responses to a 3-s sensory stimulation (olfactory, auditory, visual, tactile, and vestibular) while EDA is recorded continuously. The EDA of children with SMD recorded during the sensory challenge protocol showed an over-responsivity pattern, a larger amplitude of responses, and more responses after each stimulus (McIntosh et al., 1999). There were no differences between children with SMD and typically developing children in changes in response magnitude with repeated stimulation. However, the habituation patterns of children with SMD were slightly slower than those of the control group (McIntosh et al., 1999). Furthermore, Brown et al. (2001) found differences in the physiological responsivity and habituation of adults with different sensory patterns. Specifically, adults with SOR patterns were more responsive than the low-registration and sensation-seeking groups. People with SOR also needed more trials to habituate than the sensation-avoiding and low-registration groups did. These findings support the exploration of slower habituation as an underlying mechanism of SOR using EDA.

We sought to examine the association between the prominent sensory questionnaire measurements and physiological responses to sensation. This was conducted as a means to validate the underlying SOR processes and the current study’s newly devised physiological experimental protocol as capturing SOR. Interestingly, questionnaire reports of behavioral responses to sensory stimulation did not consistently correspond with physiological responses to sensory stimuli. Some studies did not find significant correlations between behavioral tools used to measure SOR and reactivity variables measured by EDA (Schoen et al., 2009; Lane et al., 2012; McCormick et al., 2014). Lane et al. (2012) found that sensory self-reports were correlated with anxiety but not with physiological sensory measures. Examining the relations between self-report measures and physiological measures is always challenging (Morse, 2003). Previous studies that compared the autonomic, behavioral, and parent- or self-report SOR measures have reported mixed results. Some found no correspondence of sensory questionnaires with physiological measures (Woodard et al., 2012), whereas others found partial correspondence (Brown et al., 2001). In the current study, we chose to address the question of correspondence to physiology by looking at separate questionnaires evaluating sensitivity versus habituation, questionnaires which are also perceptually oriented.

Since this study is exploratory in its nature and examines a new protocol, we recruited for this experiment a non-clinical population. This decision relied upon a rich literature that investigated traits of psychopathology in the general population (Fullana et al., 2010; Berry and Laskey, 2012; Dar et al., 2012; Taylor et al., 2014). Some characteristics of various mental disorders are found on a spectrum in the general population. For example, anxiety, the tendency to obsess, perfectionism, and harm avoidance, are all characteristics that appear at various levels in the general population. When these characteristics reach a clinical threshold level including their interference with daily functioning, and are accompanied by additional symptoms it may indicate psychopathology. Investigating nonclinical levels of psychopathology traits enables us to identify risk factors for psychopathology. Studying correlations between SOR and other traits and behaviors that are usually found in psychopathology can highlight the likelihood for developing psychopathology or risk factors for developing it. In addition, studying these phenomena in non-clinical population is important for identifying non-treated individuals.

The current study had two main goals:

1. To validate a protocol for physiological measurement of auditory habituation relative to self-report questionnaires of SOR.
2. To examine the association between OC symptoms and habituation, as measured physiologically and behaviorally in healthy adults.

## Materials and Methods

### Participants

Using a snowball sampling method, we recruited 144 participants (60.6% female and 39.4% male) via social networks. Inclusion criteria were the absence of diagnosed mental illness and age ranging from 18 to 60 years. Participants’ ages ranged from 19 to 60 years, with a mean of 33.7 (*SD* = 10.5). Four participants (3.84%) reported medical conditions such as diabetes, hypertension, and arthritis. Most (71.2%) had a bachelor’s degree or higher. The rest of the sample had a high school education (21.2%) or other non-academic higher education (7.7%).

### Instruments

#### Sensory Questionnaires

##### Adolescent/Adult Sensory Profile (Brown and Dunn, 2002)

The Adolescent/Adult Sensory Profile (AASP) is a 60-item, self-report scale designed to measure sensory-processing style. Each item describes a behavior related to an everyday sensory experience that is rated on a 5-point Likert scale indicating how frequently the behavior is performed (5 = almost always performed to 1 = almost never performed). Each item corresponds to one of four specific sensory-processing patterns (sensory sensitivity, low registration, sensory avoidant, and sensory seeking). The SOR score was used for this study, which is a sum of sensory avoidant and sensory sensitivity scores. Higher scores indicate stronger expressions of the pattern. The questionnaire was translated into Hebrew by Parush et al. (2006).

##### Sensory Processing Questionnaire, short version (Tavassoli et al., 2014)

This 35-item self-report measure assesses basic sensory function, including hypersensitivity (28 items) and hyposensitivity (seven items), across five modalities. Sensory Processing Questionnaire (SPQ) items are rated on a Likert scale from 0 (strongly agree) to 3 (strongly disagree). For easier readability of SPQ scores, items that identified hypersensitivity were reversed, so that a higher score indicated higher SOR. A SOR summary score was computed, and principal component analysis showed that most items loaded on one factor. In the current sample, the SPQ internal reliability was high (*α* = 0.84). The questionnaire was translated into Hebrew (withheld for blind for review) with permission of the authors.

##### Sensory Habituation Questionnaire (withheld for blind review)

The Sensory Habituation Questionnaire (S-Hab-Q) is a 25-item self-report measure that assesses the sensory habituation aspect of SOR. Items are rated on a 4-point Likert scale that captures the time dimension of the habituation process: “not long at all” (0) represents a very fast habituation process, “not a very long time” (1) represents a regular habituation process, “extremely long time” (2) represents a somewhat slow habituation process, and “I can’t get used to it” (3) might imply a deficit in habituation. Higher S-Hab-Q summary scores indicate slower habituation capability. The S-Hab-Q construct validity (withheld for blind review) was previously tested relative to existing SOR scales (AASP: Brown and Dunn, 2002; SPQ: Tavassoli et al., 2014) and found satisfactory (*r* = 0.57 and 0.61, respectively, *p* < 0.001). In the current sample, the S-Hab-Q internal reliability was high (α = 0.90).

#### Obsessive–Compulsive Inventory-Revised (Foa et al., 1988)

The Obsessive–Compulsive Inventory-Revised (OCI-R) lists 18 characteristic symptoms of OCD. Each symptom is rated on a 4-point Likert scale ranging from 0 (not at all) to 4 (extremely) with regards to the symptom’s prevalence during the last month. The OCI-R has been shown to have good validity, test–retest reliability, and internal consistency in both clinical (Foa et al., 1988) and non-clinical samples (Hajcak et al., 2004). The internal consistency of the OCI-R in this study was high (Cronbach’s α = 0.91).

#### Demographic Questionnaire

This questionnaire included demographic and background questions, such as age, gender, country of birth, years of education, medical problems or diagnoses, history of psychiatric disorders, and medications consumed.

### Experiment

#### Stimuli

Studying physiological responses to auditory stimuli presents an opportunity to carefully control experimental conditions (e.g., type, intensity, and duration of stimuli).

To understand reactions to daily stimuli, we chose continuous daily auditory stimuli rather than a series of short, unrelated sounds, which usually do not represent real life. In order to select the auditory stimuli for the experiment, we relied upon an international sound repository, International Affective Digital Sounds (IADS), on which a comprehensive study was conducted by Stevenson and James (2008). The researchers introduced 111 daily sounds for which participants were asked. Investigating non-clinical levels of psychopathology traits enables us to identify risk factors for psychopathology. Studying correlations between SOR and other traits and behaviors that are usually found in psychopathology can highlight the likelihood for developing psychopathology or risk factors for developing it. In addition, studying these phenomena in non-clinical population is important for identifying non-treated individuals negative stimulus hence were not appropriate for creating two distinct conditions. For the aversive condition of the current experiment, we chose three aversive auditory stimuli (AV) that the IADS protocol rated as having a high negative arousal effect (i.e., electric buzzing, drill, and car horns). For the neutral condition of the experiment, we chose three different neutral auditory stimuli (NE) that were rated in the IADS protocol as having a neutral arousal reaction (i.e., bird chirping, a trickle of water, and piano sounds). The auditory stimuli were applied to both ears using Sony wh-1000xmx3 headphones. The experimental design was programmed using E-Prime software, and was controlled by a Lenovo laptop. Each stimulus was displayed at 35 dB for 40 s duration.

#### Skin Conductance

Physiological sensitivity and habituation patterns were assessed via sweat gland EDA, which indicates sympathetic nervous system arousal. To record EDA, we used the hardware module of the BIOPAC MP150 acquisition system. Two electrodes were placed on the distal phalanges of the index and middle fingers of the participant’s non-dominant hand and secured with a Velcro band. The sensor-sampling rate was 200 Hz.

The EDA was recorded for 9 min and, after a 10-min break, again for 9 min. The experiment’s total duration was about half an hour, which included 18 min of experiment and 10 min of break.

### Operational Definitions of Habituation and Sensitivity

#### Definition of Habituation and Sensitivity at the Self-Report Level

We used the total SPQ score to represent self-reported sensitivity and the S-Hab-Q to define self-reported sensory habituation. We also used the AASP to reflect both sensitivity and habituation because the AASP questionnaire was designed to capture both dimensions.

#### Definition of Habituation and Sensitivity at the Physiological Level

We defined *sensitization* as event-related responses that occur in skin conductance when presented with a stimulus (Boucsein, 1992). The responses were measured by the average amplitude generated after the presentation of a stimulus. High physiological sensitivity would be reflected in higher average amplitude. The method applied for calculating the response average (stimulus average) was based on a well-documented methodology applied in the field of EDA (Boucsein, 2012). We were specifically interested in skin conductance response (SCR), the phasic change in EDA – a fast change in the amplitude of the signal relative to baseline. SCRs were automatically detected and their amplitudes were quantified using Matlab software. False SCRs were removed after visual inspection of the entire signal. SCRs were associated with a specific stimulus if their onset appeared at least 1.0 s after participants were presented with a stimulus. The signal of each participant was normalized and parsed into 12 trials (i.e., a total of 12 stimuli, 3 × 2 for each condition.). The baseline of each trial was calculated as the averaged signal during the 2 s preceding the stimulus onset. The stimulus average is measured in microSiemens (μS). The stimuli amplitude of each trial was calculated as the difference between the averaged signal peaks during the stimulus presentation and the trial’s baseline.

We defined *habituation* as the difference between the average response to a stimulus relative to the baseline prior to that stimulus and the average resting score. The average resting value is calculated as the averaged signal (in μS) value at rest time, once the stimulus presentation is over. A “slow to habituate” pattern is reflected by a smaller disparity of these variables. Because the ability to habituate is the ability to return to baseline after presentation of a stimulus, an inability or a slow pattern of returning to baseline might imply slow habituation.

### Behavioral Measurement

We used the total number of key presses across the experiment (*Keypress*) to measure the behavioral reaction to the stimuli.

### Procedure and Data Analysis

**FIGURE 1:**
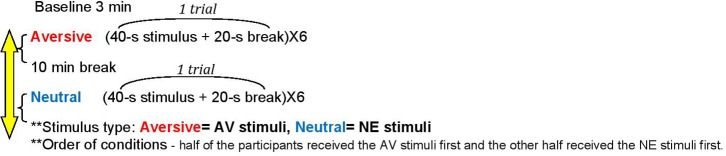
The experimental design. Stimulus type: Aversive, AV stimuli; Neutral, NE stimuli. Order of conditions – half of the participants received the AV stimuli first and the other half received the NE stimuli first.

This study was approved by the Ethics Committee of the University of Haifa (blinded for review). All participants signed an informed consent form prior to taking part in the experiment. They were told that the experiment was about sensitivity and obsession. Participants completed the self-report questionnaires online prior to the experimental phase. They were instructed to avoid physical activity, smoking, and drinking coffee during the 2 h preceding the experiment. Participants sat behind a table, in front of a computer screen. While reading the instructions for the experiment, electrodes for measuring skin conductance were placed on the participants’ phalanges. Participants were then asked to place the earphones on their heads and adjust them for optimal comfort. Following the instructions, a baseline skin conductance was recorded with no sound presentation for 3 min (see Figure 1). Afterward, two types of auditory stimuli were presented: AV and NE. The conditions were presented in counterbalance order across participants. Half the participants were presented with the AV condition first, followed by the NE condition; while the other half were presented with the NE condition first, followed by the AV (i.e., order of condition). Participants were told they could shorten the stimulus duration by pressing the space key. During each auditory stimulus, no image was displayed on the computer screen.

Each sound was presented for 40 s and followed by a 20-s break (one trial = 1 min). Each condition included three trials, each presented twice, with a total of six trials per stimulus type. If a participant pressed the space key up to 20 s from sound presentation, then the stimulus duration in that trial would shorten to 20 s. However, if a participant pressed the space key within the range of 20–40 s of stimulus presentation, then the auditory stimulus would stop immediately, and a 20-s break would follow. Between the experimental conditions, the participants had a 10-min break, during which they could drink water and use the lavatory. The total experiment time was 30 min, including the break between conditions. Participants were compensated with a gift card.

**FIGURE 2:**
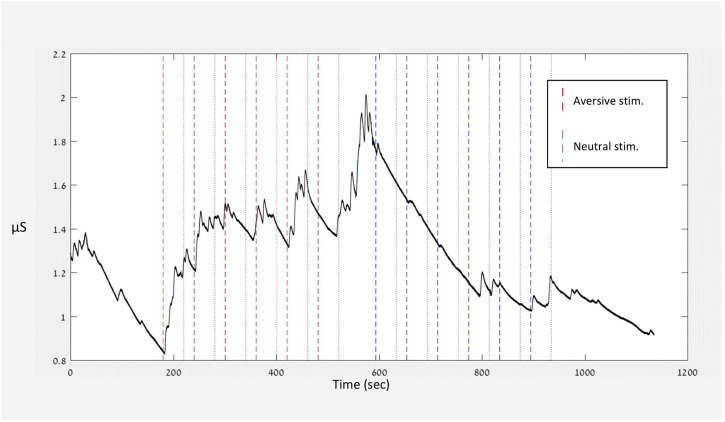
Typical recordings (raw data) of a single participant.

Figure 2 presents the raw data of one participant. The figure illustrates the experiment’s course and the participant’s specific signal along the various conditions. The given stimuli are indicated by dashed lines throughout the figure. From the signal of each participant, sensitivity (stimulus average) and habituation (stimulus average – baseline – rest average) were calculated as described in section “Operational Definitions of Habituation and Sensitivity.”

Invalid segments were marked by an automatic algorithm followed by visual inspection of the data and replaced by a linear interpolation. The EDA signal of each session was normalized and parsed into trials, time locked to the stimulus beginning. For each trial, the averaged signal during the 2 s preceding the stimuli was taken as the trial baseline and subtracted from the rest of the trial samples. The averaged signal was calculated during the auditory stimuli and the following break. Trials were excluded from the analysis if more than 50% of the recordings were invalid during the baseline test, during presentation of auditory stimuli, or after the break. Invalid trials were those with unreadable signals or with technical problems decoding the signal. Participants’ responses were included in the analysis only if they had at least two valid trials in each condition. In addition, any data point in a single trial above three standard deviations from the mean was considered an outlier and excluded from the study.

In total, 10 outlier trials were excluded from the study. For two more participants, the signal itself could not be decrypted due to a technical malfunction. To summarize, of the 104 participants who completed the experiment, 12 were excluded (*N* = 92).

To address our study’s first goal, linear mixed-model (LMM) analysis was used to assess the SCR outcomes within- and between-subject effects. Experiment conditions were defined as within-subject factors. We divided the sample into high/low SOR groups according to each participant’s score in each of the three sensory questionnaires relative to the sample’s median. The LMM was computed for each variable (physiological/behavioral sensitivity and habituation) and group (high/low SOR) by trial (stimuli type: AV vs NE and order of condition AV first vs NE first).

To address the second study goal, we used LMM analyses. Obsessive–compulsive symptoms (OCS) served as the between-subject effect (high vs low OCS), while the two experiment conditions (stimulus type: AV vs NE and order of condition AV first vs NE first) were defined as within-subject factors. Again, we examined the physiological and behavioral indices as defined earlier.

Due to multiple comparisons within tests, we conducted Bonferroni corrections, setting the alpha value threshold in accordance with the number of tests.

## Results

### Protocol Validation

#### Sensitivity

The LMM revealed two main effects for sensitivity – one for the stimulus type (AV vs NE) and the other for order of condition. In addition, there was a significant stimulus type × order of condition interactions, as shown in Table 1. Results show that for sensitivity, sensitivity level was different in each stimulus type, relative to the first stimulus which was presented (i.e., if the first stimulus that was presented was NE the sensitivity to the AV was lower, and vice versa).

**TABLE 1:** Condition and presentation effects, and interactions of sensitivity and habituation.

**EDA**	**Main effect stimulus type**		**Main effect order of condition**		**Order of conditions × stimulus type**	
	***F* (*p*)**	**Cohen’s *d***	***F* (*p*)**	**Cohen’s *d***	***F* (*p*)**	**Cohen’s *d***
Sensitivity	8.77 (<0.001)	0.170	7.57 (<0.001)	0.190	38.55 (<0.001)	0.40
Habituation	1.84 (0.170)		1.93 (0.160)		16.70 (<0.001)	0.034

**N* = 92. *df* for all EDA indices was 1,1109 for the main effects and 1,1108 for the interaction effects. Stimulus type: AV or NE, order of conditions: AV presented first or NE presented first.*

#### Habituation

The LMM did not reveal a main effect for habituation. A significant stimulus type × order of condition interaction was found (Table 1).

### Self-Report Measures

The sensory questionnaires were used to examine whether the levels of self-reported sensitivity and habituation corresponded to the physiological level of sensitivity and habituation. Table 2 presents the groups that were derived from the three sensory questionnaires’ scores.

**TABLE 2:** High and low scores of self-report sensory questionnaires.

**Questionnaire**	**High *M* (*SD*) *N***	**Low *M* (*SD*) *N***	***t***	***p***
SPQ	64.21 (8.42) 56	44.88 (7.66) 48	−12.16	<0.001
S-Hab-Q	21.29 (9.23) 55	5.00 (3.36) 49	−11.67	(<0.0010.001)
AASP/SOR	119.00 (7.97) 55	94.12 (11.28) 49	−13.08	<0.001

*SPQ, Sensory Processing Questionnaire; S-Hab-Q, Sensory Habituation Questionnaire; AASP/SOR, Adolescent/Adult Sensory Profile – sensory over-responsivity.*

For the physiological indices, high AASP/SOR scores had a significant effect on sensitivity during stimulus presentation (*F*1,11108 = 17.39, *p* < 0.001). A significant effect was found for self-reported sensitivity and habituation on the derivative of habituation, as presented in Table 3. The high S-Hab-Q score group, as well as the high SPQ score group, had worse physiological habituation. However, no effect was found for self-reported sensitivity and habituation on physiological sensitivity.

**TABLE 3:** Mixed models: sensory self-report, physiological sensitivity, and habituation.

**EDA**	**SPQ**		**S-Hab-Q**		**AASP/SOR**	
	***F* (*p*)**	**Cohen’s *d***	***F* (*p*)**	**Cohen’s *d***	***F* (*p*)**	**Cohen’s *d***
Sensitivity	0.810 (0.360)		0.172 (0.670)		17.390 (<0.001)	0.24
Habituation	4.340 (<0.001)	0.62	15.280 (<0.001)	0.06	23.950 (<0.001)	0.29

**N* = 92. *df* for all EDA indices was 1,1109. EDA, electrodermal activity; SPQ, Sensory Processing Questionnaire; S-Hab-Q, Sensory Habituation Questionnaire; AASP/SOR, Adolescent/Adult Sensory Profile – sensory over-responsivity.*

For the AASP/SOR scores, we found a significant effect in the *Keypress*, the behavioral measures of the protocol – shortening the duration of the stimulus and pressing for shortening before 20 s of stimulus presentation. High AASP/SOR scores were related to the *Keypress* score (*F*1,126 = 8.61, *p* < 0.001).

### Behavioral Measure

In most trials (80% AV and 97% NE), the participants did not press the space key – choosing to listen to the stimulus for 40 s. However, significant differences (χ^2^ = 83.69, *p* < 0.001) were found in the number of *Keypress* in the AV condition compared to the NE condition. In 84 (15%) of the AV condition trials, participants executed “key presses” in the range between the onset of stimulus presentation and 20 s, and 5% *Keypress* were executed 20 to 40 s after stimulus presentation. Participants chose to shorten the stimulus length in only 3% of the NE condition trials.

### Habituation/Sensitivity and OCS

#### Self-Reported Sensitivity/Habituation and OCS

Significant correlations were found between self-reported OCS and all sensory self-report measures (*N* = 104, *p* ≤ 0.006). The OCI scores correlated with SPQ (*r* = 0.42), S-Hab-Q (*r* = 0.51), and AASP/SOR scores (*r* = 0.48).

To compare the physiological habituation and sensitivity of individuals with high versus low OCS, two OC groups were assembled from our sample of healthy adults. The total OCI scores were used to identify 15 low-scoring (below 14) OC participants and 21 high-scoring (above 24) OC participants (Foa et al., 1988). The high OCS group included four males (19%) and 17 (81%) females. The ratio between male and females was significantly reversed in the low OCS group, which was comprised of 11 males (73.3%) and four females (26.7%).

Independent sample *t*-tests showed that participants with high OCS were younger compared to the low OCS group (Table 4).

**TABLE 4:** Characteristics comparison of high- and low-obsessive–compulsive symptoms (OCS) groups.

**Variable**	***M* (*SD*)**		***t***	***p***
	**High OCS (*n* = 21)**	**Low OCS (*n* = 15)**		
Age, years	28.80 (10.40)	37.50 (8.30)	4.82	<0.001
OCI-R	31.90 (6.38)	5.40 (2.09)	4.40	<0.001
S-Hab-Q	26.95 (8.49)	2.60 (2.41)	6.66	<0.001
SPQ	61.86 (11.72)	49.50 (11.37)	8.58	<0.001
AASP/SOR	98.43 (12.32)	117.00 (12.11)	19.14	<0.001

*OCI-R, Obsessive–Compulsive Inventory-Revised; S-Hab-Q, Sensory Habituation Questionnaire; SPQ, Sensory Processing Questionnaire; AASP/SOR, Adolescent/Adult Sensory Profile – sensory over-responsivity. Following a Bonferroni correction, the *p* value threshold was set to 0.002.*

#### Physiological Measures and OC Tendencies

##### Sensitivity

Interaction effects were found between OCS and stimulus type (AV vs NE) relative to sensitivity (Table 5). A second interaction was found between OCS, stimulus type, and order of condition.

**TABLE 5:** Mixed models: condition, presentation, and obsessive–compulsive symptoms (OCS) effects and interactions.

**EDA index**	**Stimulus type × OCS**		**Order of condition × OCS interaction**		**Order of condition × stimulus type × OCS**	
	***F* (*p*)**	**Cohen’s *d***	***F* (*p*)**	**Cohen’s *d***	***F* (*p*)**	**Cohen’s *d***
Sensitivity	5.89 (0.015)	0.001	0.64 (0.421)		7.02 (<0.001)	0.023
Habituation	1.58 (0.209)		7.81 (0.005)	0.07	5.34 (0.005)	0.11

**df* for sensitivity was 1,615 for the two factors interaction and 2,615 for the three factors interaction, *df* for habituation was 1,583 for the two factors interaction and 2,583 for the three factors interaction. Stimulus type: AV or NE, order: AV presented first or NE presented first.*

*Post hoc* analysis revealed that the effect was due to differences between the SCR signal of the high and low OCS: high OCS had significantly higher sensitivity to AV stimuli in all Orders of conditions, compared to the low OCS group (*p* = 0.038), and compared to the high OCS group’s reaction to the NE stimuli (*p* < 0.001). The high OCS group had the same sensitivity reaction to the AV stimulus in all orders of conditions (*p* = 0.16), they did not have a significantly higher sensitivity when the aversive condition was presented first (*p* = 0.82).

**FIGURE 3:**
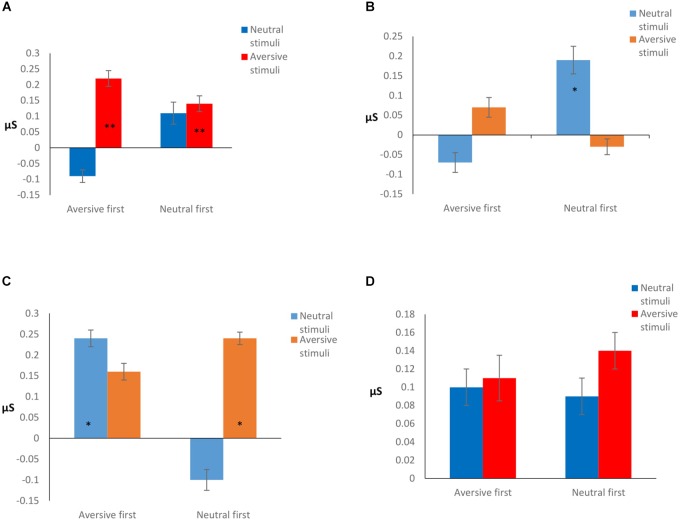
**(A)** High OCS sensitivity. **Significantly higher reaction compared to low OCS aversive stimuli (*p* = 0.038), and compared to high OCS reaction to the neutral stimuli (*p* < 0.001). **(B)** Low OCS sensitivity. *Significantly higher reaction to the neutral stimulus when it was presented first (*p* = 0.014), compared to the low OCS reaction to the aversive stimuli in the same condition. **(C)** Low OCS habituation. **(D)** High OCS habituation. Habituation is calculated as the difference between the stimulus average and the rest average after stimulus presentation. The higher the difference, the better the habituation. *Significantly different in habituation between conditions (*p* = 0.035), and stimulus type (*p* = 0.001).

Low OCS showed a significantly different reaction between conditions: a higher reaction to the NE stimulus when it was presented first (*p* = 0.014) but no differences between the reaction to the AV stimuli (*p* = 0.12). However, the low OCS had no significant differences in the value of the NE stimulus response compared to the high OCS (*p* = 0.16). Results are presented graphically in Figures 3A,B.

#### Habituation

The LMM did not reveal a main effect of group for habituation (*F*1,583 = 1.38, *p* = 0.24); however, under the experimental conditions the groups reacted differently in terms of habituation. Interactions of OCS × order of condition and a three-factor interaction of OCS × stimulus type × order of condition were found (presented in Table 5).

*Post hoc* analysis showed that when AV stimuli were presented first, low OCS had a better habituation to the NE stimulus (*p* = 0.035), while the high OCS had no differences in their habituation patterns to NE stimulus presented after an AV stimulus (*p* = 0.34). When the NE stimulus was presented first, the low OCS had a better habituation to the AV stimulus (*p* = 0.001), while the high OCS had no differences in their habituation patterns to AV stimulus presented after a neutral stimulus (*p* = 0.25). Results are presented graphically in Figures 3C,D.

### Behavioral Measures and OC Tendencies

The LMM revealed a main effect of stimuli type for *Keypress* (*F*1,692 = 50.81, *p* < 0.001), and interaction for group × stimuli type (*F*1,692 = 15.81, *p* = 0.005).

*Post hoc* analysis showed that the high OCS had shortened the stimuli presentation by pressing a key significantly more times than the low OCS group (*p* < 0.001), and were more prone to do so for the AV stimuli (*p* < 0.001).

## Discussion

This study aimed to understand the association between elevated OC symptomatology and two underlying facets of SOR, sensitivity and habituation. As such, the study presents the design and validation of an experimental protocol measuring auditory habituation and sensitivity in adults at the physiological and behavioral levels. The primary results reveal that OCS correlate with self-reported SOR and those adults with high OCS show slower habituation patterns compared to those with low OCS. With regards to protocol validation, the main findings were that the interaction between stimuli type, and the order of condition (in this experiment, aversive first or neutral first) had influenced the habituation process while the stimuli type by itself had no effect on habituation. Self-reported SOR was more related to physiological habituation than to physiological sensitivity.

### Protocol Validation

In terms of protocol validation, we found that both the stimulus type (AV or NE) and order of condition affected sensitivity. As expected participants reacted with greater sensitivity to the AV condition than to the NE condition. The stimulus type which was previously presented effected the level of reactivity in the following stimuli.

When looking at habituation, we found the stimulus type and order of condition to be the parameters that determine the ability to habituate faster. Regardless of the stimulus type, habituation was always faster when the second *condition* presented. Despite our initial hypothesis that it is more difficult to habituate to a stimulus that evokes more reactivity or is more unpleasant, in practice previous presentation of an auditory stimulus had a greater impact on auditory habituation than did the type of stimulus.

Other EDA studies testing SOR in various populations also found a significantly decreased response between the first trials (regardless of sensory stimulus type) and those that followed (Schoen et al., 2009; McCormick et al., 2014). Our results show that participants had a tendency toward higher reactivity to, and demonstrated slower habituation patterns in, the AV condition. However, these results had no statistical significance. The type of stimulus was less determinant of the physiological response while the order of conditions did. Although statistically the type of stimulus did not affect habituation or reactivity, it did have some effect on these parameters. Our results cannot be directly compared with others as previous EDA sensory studies (e.g., McIntosh et al., 1999; Schoen et al., 2009) had not considered the different valences each stimulus has or their possible influence on EDA; other studies used the same auditory stimuli repeatedly (Van Engeland, 1984).

The non-significant results of the stimuli type effect in the current study could be due to the specific type of stimuli chosen. Our EDA protocol includes a few unique components: (a) a classification of the stimuli as having an aversive or neutral effect on the listener, and (b) a longer duration for which each stimulus was displayed (continuous stimulus).

Anecdotally, after completing the experiment, some participants reported that both conditions were equally unpleasant; others reported that the NE condition was even more bothering than the AV one. If SOR is characterized by an abnormal reaction to normal everyday stimuli (Dunn, 1997) – that is, the response may be the same whether it is a neutral or an aversive stimulus – then how can the differences between conditions be explained? We believe part of the explanation lies in the presentation *duration* of each stimulus. This experiment’s uniqueness is that each stimulus lasted 40 s, as opposed to 3 s in other protocols (e.g., McIntosh et al., 1999). Thus, part of the decrease occurred simultaneously while the stimulus was being played. We conjecture that this affects the ability to habituate, just as in real life, where stimuli are ongoing, and one must acclimate to them. It is possible that when a stimulus that has a negative effect is displayed for a few seconds, the habituation process is slower, perhaps also due to attentional bias to negative input (e.g., Smith et al., 2003).

As mentioned earlier, the order of condition factor was the only parameter that affected habituation. This finding has significant clinical implications for both those with SOR and those with OCD. Repeated exposures, as well as long exposures to unpleasant stimuli, are required to help the sensory system acclimate and reduce sensitivity, anxiety, and avoidance reactions throughout development. Another clinical significance of this could be for the design of intentional training in which different-effect stimuli are given intermittently.

### Cross-Measurement of SOR

One aim of the current study was to test the correspondence of physiological measures with the more commonly used self-reporting SOR measures. Physiological sensitivity corresponded only with the AASP/SOR score but not with the SPQ or the S-Hab-Q. This is a surprising finding, in part because the SPQ was designed to capture the sensitivity dimension and specifically the ability to detect stimuli. The S-Hab-Q was not designed to capture sensitivity; thus, it is not surprising that it did not correspond with physiological sensitivity. These inconsistent correlations between self-reported SOR and physiological measures are in line with other studies (Woodard et al., 2012). Two alternative interpretations can explain the interaction between high physiological sensitivity and high AASP/SOR. The first is that the AASP indeed measures the sensitivity aspect of SOR and therefore also corresponds with physiological sensitivity. That is, people who reported themselves as having higher sensory sensitivity also had higher physiological sensitivity. Another possible explanation is that the AASP contains items that are more behavioral and emotional in nature compared to the other perception-oriented sensory questionnaires we used. The SOR scores of the AASP correlate with anxiety and arousal levels (Engel-Yeger and Dunn, 2011), which affect EDA (Fowles, 1980). Hence, anxiety is expressed by elevated physiological reactivity but is not necessarily a specific indicator of SOR. These findings should encourage clinicians to use more than one approach to diagnose and assess SOR. A feasible diagnostic battery that includes behavioral, physiological, and self-report measures is needed to better diagnose and evaluate SOR.

In contrast to the limited correspondence of self-report with physiological sensitivity, all questionnaires used in this study related with physiological habituation. It is possible that the mechanism underlying SOR is a deficit in habituation and not high sensitivity. Due to the small effect of the findings (Cohen, 1988) this proposed mechanism should be further investigated carefully. Some researchers claimed that the neurological impairment underlying the symptoms of SOR is a deficit in SG (Miller et al., 2009); in other words, an inhibition deficit that prevents habituation. Although EDA does not reflect SG, in our understanding there is a similarity between the theoretical structure of SG and habituation, as was measured through EDA indices. Incorporating SG measures in future research is warranted to further test the habituation mechanism of SOR in relation to OC put forward by this study. The different patterns of association between physiological and self-reported habituation versus sensitivity support their distinction. These findings can justify the future use of separate questionnaires for each construct, habituation and sensitivity, as well as continued research into each one’s unique contribution to SOR and related difficulties.

### OCS and SOR

Individuals in this study with elevated OC traits were prone to report high levels of sensitivity and habituation, but their self-reported sensory questionnaire results differed significantly from their physiological patterns. This finding is consistent with previous reports of high correlations between self-reported SOR and OCS in healthy adults (Dar et al., 2012; Taylor et al., 2014; Ben-Sasson and Podoly, 2017). The difference between self-reported sensory questionnaire and physiological measures that was found in the current study is also consistent with other studies that found that reporting sensitivity does not necessarily imply actual physiological sensitivity (Belluscio et al., 2011; Güçlü et al., 2015). In order to understand whether the results support the relation between habituation and OC symptoms, results must be examined in several contexts.

The SOR–OCD correlations have received various explanations: SOR as a trait marker for a specific OCD subtype (Summerfeldt, 2004; Ferrão et al., 2012), SOR as a vulnerability factor for developing psychopathology in general (Levit-Binnun et al., 2013; Conelea et al., 2014), or SOR as a developmental sensory basis for determining pathological cognitive schemes (Summerfeldt, 2004). Nevertheless, SOR should be evaluated in OCD as part of the diagnostic procedure, due to its great impact on the severity of psychopathology (Conelea et al., 2014) and on quality of life (Bar-Shalita et al., 2008).

Both self-reported sensitivity and self-reported habituation correlated with OCS, indicating it is not possible to deduce a distinct common mechanism that links sensory habituation (as opposed to sensitivity) to OCD and OCS. However, none of the sensory questionnaires, including the ASAP, had a significantly stronger correlation with the OCI. A justification for separating questionnaires for sensitivity and habituation might come from the physiological results, which showed that high-OCS group took longer to habituate to the stimuli but did not differ in their reactivity levels. This might imply that sensory habituation, that is specifically measured physiologically, has an important role in OCD. It is possible that the self-reported questionnaires of sensitivity and habituation did not differ substantially from one another, and from the ASAP questionnaire. Therefore, a stronger correlation between one of the dimensions and OC was not found, however, this does not imply that we should not evaluate these dimensions in clinical context, especially since they have some implication on practice, as we will describe later on.

When looking at the physiological results, the high OCS group seemed to have the same habituation rates regardless of stimuli type or order of condition, which means that instead of acclimating, they continued to respond to the stimulus at the same manner. Although the sensitivity of the high OCS to the AV stimuli was higher when compared to the low OCS, overall they did not show more physiological sensitivity. These findings could be due to an attention bias toward stimuli that would normally be processed without conscious awareness (Buse and Roessner, 2016) or a result of a slow habituation process (Hallett, 2015). In a recent review by Thielen and Gillebert (2019) a few factors that influence sensory sensitivity are described. Among these factors are the predictability of a stimuli, its’ relevance to a specific and current goal, or in other words – motivation and attention. This study’s findings in which high OCS did not use prior stimulus as a cue to modulate the habituation of the following stimulus, can be explained by an inadequate prediction. The inadequate prediction model has been applied to explain atypical sensory sensitivity in various clinical populations, such as autism (e.g., Pellicano and Burr, 2012; van de Cruys et al., 2014). Our findings suggest that habituation can serve as a shared mechanism for explaining SOR’s interplay with OCS.

### Limitations and Directions for Future Research

One limitation of this study is its sample’s lack of representation and small size. Although the literature describes an equal ratio of males and females with OCD, our sample was unequal in gender (and age) representation. A higher number of younger and female participants were represented in the high-OCS group than in the low-OCS group. This gender inequality may have resulted from recruitment within university programs where there is a female dominance. This bias also occurred in a previous study, in which the high-OCS group had a higher ratio of females versus males and a lower mean age (Lazarov et al., 2010). Another limitation is the use of a non-clinical sample, which does not allow direct deduction to a clinical sample.

Choosing EDA to measure auditory habituation might have also affected our data. Although EDA is a measure of the arousal system, it does not directly measure sensory processing, sensory sensitivity, or habituation. However, skin conductance is a reliable measure of arousal and reactivity (Miller et al., 2009; McCormick et al., 2014).

Habituation is an SOR dimension that is not yet fully understood and treated in research and intervention. This study examined both sensitivity and habituation as two separate dimensions of SOR. Future studies using a multi-methods approach (i.e., self-report, physiological, and behavioral measures) will help to quantify SOR and clarify the neurological mechanisms underlying these observable behaviors. We suggest that future research make use of more robust physiological measures such as SG to capture these SOR dimensions. We also recommend examining attention bias as a competing factor that can affect the habituation process.

This study examined only the auditory modality. An examination of all sensory modalities is necessary to obtain a comprehensive picture of habituation and sensitivity. In addition, to establish understanding of the co-appearance of SOR and OCS, the protocol should be examined with a clinical OCD sample as well as those with other clinical conditions associated with SOR, such as with anxiety and schizophrenia.

## Conclusion

We introduced a multi-method study design that used both self-report and physiological measures to examine SOR dimensions. Using different measurement methods presents a significant challenge: self-report did not consistently correspond with physiological measures in differentiating groups and SOR constructs. However, we believe that by combining different measurements, a more accurate and reliable assessment of SOR can be achieved. Differences were found between the sensitivity and habituation patterns of healthy adults with high versus low OCS. Differentiating between habituation and sensitivity has diagnostic and therapeutic implications. This study calls for further examination of the topic, with different physiological indices and clinical populations.

## Data Availability Statement

The datasets generated for this study are available on request to the corresponding author.

## Ethics Statement

The studies involving human participants were reviewed and approved by the Faculty of Social Welfare and Health Sciences, University of Haifa. The patients/participants provided their written informed consent to participate in this study.

## Author Contributions

TP and AB-S together took lead in designing the study, conducted the statistical analyses, and wrote the manuscript. TP was involved in data collection. Both authors read and approved the manuscript.

## Conflict of Interest

The authors declare that the research was conducted in the absence of any commercial or financial relationships that could be construed as a potential conflict of interest.
