# Corrigendum: Sensory Habituation as a Shared Mechanism for Sensory Over-Responsivity and Obsessive–Compulsive Symptoms

**DOI:** 10.3389/fnint.2020.00032

**Published:** 2020-06-30

**Authors:** Tamar Y. Podoly, Ayelet Ben-Sasson

**Affiliations:** ^1^Department of Occupational Therapy, Faculty of Social Welfare and Health Sciences, University of Haifa, Haifa, Israel; ^2^Cognetica: The Israeli Center for Cognitive Behavioral Therapy, Tel Aviv, Israel

**Keywords:** sensory, habituation, OCD, adults, electrodermal activity

In the original article, the authors were incorrectly ordered as Ayelet Ben-Sasson and Tamar Y. Podoly. The correct order is Tamar Y. Podoly and Ayelet Ben-Sasson.

The authors apologize for this error and state that this does not change the scientific conclusions of the article in any way. The original article has been updated.

